# The mitochondrial DNA-cGAS-STING axis in colorectal cancer: a focused review of context-dependent roles and therapeutic opportunities

**DOI:** 10.3389/fmolb.2026.1939425

**Published:** 2026-09-11

**Authors:** Huayang Gui, Yali Zhang, Xingxing Yuan

**Affiliations:** Department of Gastroenterology, Heilongjiang Academy of Traditional Chinese Medicine, Harbin, Heilongjiang, China

**Keywords:** cGAS-STING, colorectal cancer, immunotherapy, innate immunity, mitochondrial DNA, mitochondrial stress, tumor microenvironment

## Abstract

Immune checkpoint blockade produces durable benefit mainly in microsatellite instability-high/mismatch repair-deficient (MSI-H/dMMR) colorectal cancer (CRC), whereas most microsatellite-stable/mismatch repair-proficient (MSS/pMMR) tumors remain poorly inflamed and resistant to immunotherapy. This focused narrative review examines how mitochondrial DNA (mtDNA)-derived danger signals engage the cGAS-STING pathway in CRC, distinguishes canonical DNA sensing from noncanonical STING activation and STING-independent cGAS functions, and evaluates therapeutic strategies that modulate this axis. We qualitatively synthesize peer-reviewed mechanistic and translational studies addressing mtDNA release, pathway routing, cellular context, therapeutic targeting, and biomarkers in CRC, while drawing on pan-cancer and DNA-damage studies only when they clarify pathway architecture or translational constraints. No new experimental, patient-level, or case-series data are presented. Current evidence indicates that biological outcome depends on more than pathway activation alone. Signal amplitude and duration, STING trafficking and proteostasis, downstream IRF3- versus NF-κB-biased signaling, autophagy and mitophagy, metabolic state, pathway abundance, and responding cell type collectively determine whether activation supports antitumor immunity or chronic inflammation and immune suppression. Nuclear DNA damage can also activate noncanonical STING programs independently of cGAS, whereas cGAS can exert STING-independent functions. Upstream mtDNA-origin interventions may provide a more localized route to pathway activation by exploiting tumor mitochondrial stress, but cGAS is not intrinsically mtDNA-specific and any safety advantage over direct STING agonism remains unproven clinically. Clinical translation should therefore prioritize origin-resolved biomarkers, cell-specific pharmacodynamic readouts, tumor-localized and transient pathway modulation, and biomarker-stratified trials.

## 1 Introduction

Colorectal cancer (CRC) is the third most commonly diagnosed malignancy and the second leading cause of cancer-related death worldwide (Bray et al., 2024). Immune checkpoint blockade has produced durable clinical benefit in patients with microsatellite instability-high (MSI-H)/mismatch repair-deficient (dMMR) CRC. Pembrolizumab has demonstrated sustained efficacy in this population (André et al., 2020), and nivolumab-based combination therapy has further confirmed the responsiveness of dMMR/MSI-H CRC to immune checkpoint inhibition (Overman et al., 2018). In contrast, most microsatellite-stable (MSS)/mismatch repair-proficient (pMMR) tumors remain poorly responsive to immunotherapy because of limited tumor antigenicity, insufficient cytotoxic T-cell infiltration, inefficient antigen presentation, and an immunosuppressive stromal environment (Ganesh et al., 2019). Restoring effective endogenous antitumor immunity in these tumors therefore remains a major therapeutic challenge. The cGAS-STING pathway has attracted considerable interest in this context because it links cytosolic DNA sensing to innate and adaptive immune responses. Cytosolic double-stranded DNA activates cyclic GMP-AMP synthase (cGAS), leading to cyclic GMP-AMP (cGAMP) production (Ablasser and Chen, 2019). cGAMP subsequently binds to stimulator of interferon genes (STING) and initiates downstream immune signaling (Kwon and Bakhoum, 2020). The structural basis of cGAS activation, STING trafficking, and downstream signal transduction has been extensively characterized (Hopfner and Hornung, 2020). Among the potential sources of cytosolic DNA, mitochondrial DNA (mtDNA) is particularly relevant to cellular stress. Disruption of mitochondrial membrane integrity, nucleoid stability, or quality-control mechanisms can expose mtDNA to the cytosol and convert mitochondrial injury into an immunostimulatory signal (Riley and Tait, 2020). Mitochondrial genome instability can further trigger innate immune responses, thereby linking mitochondrial stress to immune recognition (West et al., 2015).

CRC provides a distinctive biological context for mtDNA-dependent immune signaling because oxidative stress, metabolic reprogramming, altered mitochondrial dynamics, and the intestinal microbial environment can all influence mitochondrial homeostasis and inflammatory responses. Cancer-associated mtDNA alterations also include mutations, epigenetic changes, and potentially targetable mitochondrial abnormalities, indicating that the mitochondrial genome itself represents an important component of tumor biology (Shetty et al., 2026). Previous reviews have established a broad mechanistic framework linking mtDNA release to cGAS-STING activation across multiple cancer types, although CRC-specific molecular subtypes and cellular contexts have received comparatively limited attention (Xia et al., 2025). More recent analyses have extended this framework by integrating mitochondrial metabolism with canonical and noncanonical STING signaling, but their scope remains largely pan-tumor (Zhao et al., 2026a). Despite these advances, the specific relationship among mtDNA origin, pathway routing, cellular identity, and therapeutic response in CRC remains insufficiently resolved. This distinction is important because increased cGAS-STING activity does not necessarily indicate mtDNA-driven signaling, particularly in settings where nuclear DNA damage or alternative STING activation routes may coexist.

Against this background, the present article provides a focused narrative review of the mtDNA-cGAS-STING axis in CRC. We examine the sequence from mtDNA generation and release to cytosolic sensing, canonical and noncanonical signal routing, cell-dependent biological effects, therapeutic modulation, and translational monitoring. Particular attention is given to the mechanisms that determine whether pathway activation supports antitumor immunity or instead contributes to chronic inflammation, immune suppression, and treatment resistance. Nuclear-DNA-derived signaling and noncanonical STING activation are discussed as mechanistic comparators where necessary to distinguish them from mtDNA-origin signaling. Evidence is drawn primarily from peer-reviewed CRC studies, with findings from other tumor types or general DNA-damage biology included when they clarify pathway architecture or translational constraints. This review presents no new experimental or patient-level data and does not undertake quantitative evidence pooling. Instead, it integrates current mechanistic and translational evidence to define the context-dependent roles of the mtDNA-cGAS-STING axis, evaluate emerging therapeutic strategies, and identify the major requirements for clinical translation.

## 2 Biological basis of the mtDNA-cGAS-STING signaling axis

### 2.1 mtDNA release and its regulation

Human mtDNA is a circular, double-stranded genome of approximately 16.6 kilobase pairs (kb) that primarily encodes subunits of oxidative phosphorylation complexes, transfer RNAs (tRNAs), and ribosomal RNAs (rRNAs) (Anderson et al., 1981). Under physiological homeostasis, mtDNA is packaged with mitochondrial transcription factor A (TFAM) into mitochondrial nucleoids and sequestered within the mitochondrial matrix. Mitochondrial damage, altered membrane permeability, or impaired quality control can disrupt this compartmentalization and expose mtDNA to the cytosol (Riley and Tait, 2020).

Mitochondrial membrane integrity represents an important barrier to mtDNA release. Loss of PLSCR3 in CRC cells disrupts inner mitochondrial membrane organization and promotes mtDNA translocation into the cytosol (Ling et al., 2026). Programmed cell-death pathways can produce a similar effect by altering mitochondrial permeability. The N-terminal fragment of GSDME induces mitochondrial damage and an mtDNA-dependent IFN-β response (Luo et al., 2024), whereas BCL-2 inhibition promotes VDAC1 oligomerization, mtDNA release, and STING-dependent antitumor immunity (Zhang W. et al., 2025). Mitochondrial injury can therefore function not only as a mechanism of tumor-cell death but also as a source of innate immune stimulation.

The organization and epigenetic state of the mitochondrial genome also influence its cytosolic availability. Deficiency of short-chain acyl-CoA dehydrogenase (ACADS/SCAD) increases mtDNA methylation and suppresses cytosolic translocation, whereas restoration of ACADS/SCAD expression promotes mtDNA demethylation and release, thereby enhancing cGAS-STING signaling (Yang F. et al., 2026). TFAM stability provides another regulatory layer. The lncRNA 606938-TFAM axis modulates TFAM protein stability through processes associated with oxidative phosphorylation and reactive oxygen species (ROS), thereby affecting mtDNA leakage (Du et al., 2026). These findings indicate that both mitochondrial metabolism and nucleoid organization can regulate the cytosolic availability of mtDNA.

Mitochondrial dynamics become particularly relevant under therapeutic stress. Targeting dynamin-related protein 1 (DRP1) can restore radiotherapy-induced mitochondrial stress in KRAS-mutant CRC and promote mtDNA translocation into the cytosol (Tsai et al., 2026). These observations extend the regulation of mtDNA release from membrane permeability and nucleoid organization to organelle dynamics. More broadly, mtDNA leakage is increasingly recognized as a link between mitochondrial injury and antitumor immune signaling (Aloraini, 2025).

### 2.2 Canonical mtDNA-cGAS-STING signal transduction and regulation

After entering the cytosol, mtDNA binds to cGAS, which uses adenosine triphosphate (ATP) and guanosine triphosphate (GTP) to synthesize the second messenger 2′,3′-cyclic GMP-AMP (2′,3′-cGAMP) (Ablasser and Chen, 2019). Structural studies have defined the molecular basis of DNA recognition by cGAS and conformational changes associated with STING activation (Zhang et al., 2020). Binding of 2′,3′-cGAMP to endoplasmic-reticulum-associated STING promotes its trafficking toward the Golgi apparatus and recruitment of TANK-binding kinase 1 (TBK1). TBK1 subsequently phosphorylates STING and interferon regulatory factor 3 (IRF3), leading to IRF3 nuclear translocation and type I interferon (IFN-I) production (Hopfner and Hornung, 2020).

STING can also engage inhibitor of κB kinase (IKK-NF-κB) signaling and promote the transcription of inflammatory mediators (Kwon and Bakhoum, 2020). The balance between IRF3-associated IFN-I signaling and NF-κB-associated inflammatory signaling contributes to the context-dependent biological effects of pathway activation.

STING abundance and proteostasis provide an additional level of regulation. In CRC, inhibition of valosin-containing protein (VCP/p97) stabilizes endoplasmic-reticulum-retained STING and enhances downstream signaling (Zhu H. et al., 2025). This mechanism acts at the level of STING protein turnover and is mechanistically distinct from increasing upstream pathway input through mtDNA release.

### 2.3 Noncanonical STING activation and STING-independent cGAS functions

Canonical mtDNA signaling begins when cytosolic mtDNA engages cGAS, leading to 2′,3′-cGAMP production and subsequent STING activation. Noncanonical STING signaling, however, encompasses mechanistically distinct routes and should not be treated as synonymous with cGAS-independent activation. For example, cGAMP-bound STING can engage protein kinase R-like endoplasmic reticulum kinase (PERK) and activate a PERK-eIF2α-dependent translational program outside the conventional TBK1-IRF3 transcriptional response (Zhao et al., 2026a).

Nuclear DNA damage provides a distinct form of noncanonical STING signaling. Following etoposide-induced DNA damage, STING activation can occur independently of both cGAS and cGAMP (Dunphy et al., 2018). ATM and PARP1 are required for the formation of an alternative STING signaling complex involving IFI16, p53, and TRAF6. IFI16 facilitates TRAF6 recruitment to STING, followed by TRAF6-dependent K63-linked ubiquitination of STING. This signaling state preferentially promotes NF-κB activity rather than the IRF3-dominant response typically associated with canonical cytosolic DNA sensing.

This pathway is not completely IRF3-independent. Low-level STING trafficking and TBK1-IRF3 activity may still contribute to IFN-β production, whereas NF-κB-dependent outputs such as IL-6 can proceed largely without TBK1 activity (Dunphy et al., 2018). Noncanonical STING activation should therefore be regarded as a qualitatively different signaling architecture rather than simply a weakened form of canonical signaling.

STING-independent functions of cGAS constitute a separate mechanistic category. cGAS can interact with the autophagy regulator Beclin 1 and promote autophagic DNA clearance independently of STING (Zhao et al., 2026b). In CRC, cGAS has also been linked to metabolic reprogramming through a STING-independent mechanism (Wang et al., 2024). These functions should not be interpreted as mtDNA-driven STING activation. The mechanistic framework used throughout this review therefore distinguishes canonical mtDNA-cGAS-cGAMP-STING signaling, noncanonical STING signaling, and STING-independent cGAS functions.

### 2.4 Determinants of context-dependent STING signaling

The biological effects of the mtDNA-cGAS-STING axis cannot be predicted simply from whether the pathway is activated. Signal amplitude and duration influence the magnitude and persistence of the response, whereas activation route, post-translational regulation, cellular identity, and metabolic state determine how that signal is interpreted.

Transient or appropriately controlled STING activation can support IFN-I production, dendritic-cell maturation, antigen presentation, and adaptive antitumor immunity. Excessive or persistent activation may instead produce immune-cell toxicity, tolerance, or chronic inflammatory signaling. This non-linear relationship is also relevant to therapeutic STING stimulation. Lower intratumoral agonist exposure may support systemic T-cell responses, whereas stronger stimulation can compromise immune activation through adverse effects on dendritic cells and T cells. Activation route and post-translational regulation further influence downstream output. Canonical cGAMP-dependent STING signaling can engage both TBK1-IRF3 and NF-κB, whereas alternative signaling states may favor NF-κB-dominant responses. ULK1-mediated phosphorylation of STING at S366 provides an example in which IRF3-IFN-I signaling can be suppressed while NF-κB activity is retained. These observations support a context-dependent model but do not establish a universal TBK1-versus-IKK molecular switch (Lu et al., 2026).

Branch dominance is more plausibly determined by the combined effects of STING trafficking, phosphorylation, ubiquitination, kinase availability, feedback regulation, and cellular context. The same pathway input may therefore generate different transcriptional programs according to the signaling architecture present in a given cell.

Mitochondrial quality control and cellular metabolism further regulate pathway persistence. Efficient mitophagy removes damaged mitochondria and limits continued mtDNA leakage, whereas defective mitochondrial clearance can prolong inflammatory signaling. PINK1-associated mitophagy and TBK1-dependent regulation of autophagy machinery provide mechanisms through which mitochondrial quality control can restrain pathway activity. Metabolic state also affects signaling competence because glycolytic activity, mitochondrial ROS, nutrient availability, and cellular bioenergetics can modify STING-dependent responses in both tumor and immune cells (Zhao et al., 2026b).

Cell identity integrates these regulatory layers. Dendritic cells can convert controlled STING activation into antigen presentation and lymphocyte recruitment, whereas excessive stimulation can compromise their survival. Macrophage responses are strongly influenced by metabolic polarization, and T cells may respond differently to cell-intrinsic STING activation depending on their activation and metabolic state. Consequently, neither cytosolic mtDNA abundance nor total STING expression alone is sufficient to predict biological outcome.

### 2.5 Cell type-dependent effector outputs

Cell identity is a major determinant of the biological consequences of cGAS-STING activation. In dendritic cells and other antigen-presenting cells, type I interferons promote maturation and cross-presentation and induce chemokines such as CXCL9 and CXCL10, thereby facilitating the recruitment of CD8^+^ T cells and natural killer (NK) cells (Kwon and Bakhoum, 2020). This transmission from innate sensing to adaptive immunity represents an important mechanism through which STING can support antitumor responses.

Pathway activation can also interact with regulated tumor-cell death. STING activation in immune cells can promote ferroptosis in CRC cells and enhance local antitumor effects (Ding et al., 2026). Conversely, tumor-cell ferroptosis induced by DNA-dependent activator of IFN-regulatory factors (DAI) can reprogram tumor-associated macrophages toward an antitumor phenotype (Cheng et al., 2025). These observations illustrate how tumor-cell death and immune-cell state can reinforce one another within the tumor microenvironment.

The consequences differ in normal or inflamed intestinal epithelium. cGAS-STING-mediated pyroptosis in epithelial cells can aggravate tissue injury and inflammation and promote inflammation-associated CRC (Wang et al., 2026a). Sustained mtDNA leakage in mitochondrial disease provides additional evidence that persistent mitochondrial danger signaling can maintain chronic inflammatory responses (Szabo et al., 2026). STING signaling also interacts with ferroptosis (Lin X. et al., 2026) and with inflammasome- and pyroptosis-associated pathways (Liu et al., 2024).

**FIGURE 1:**
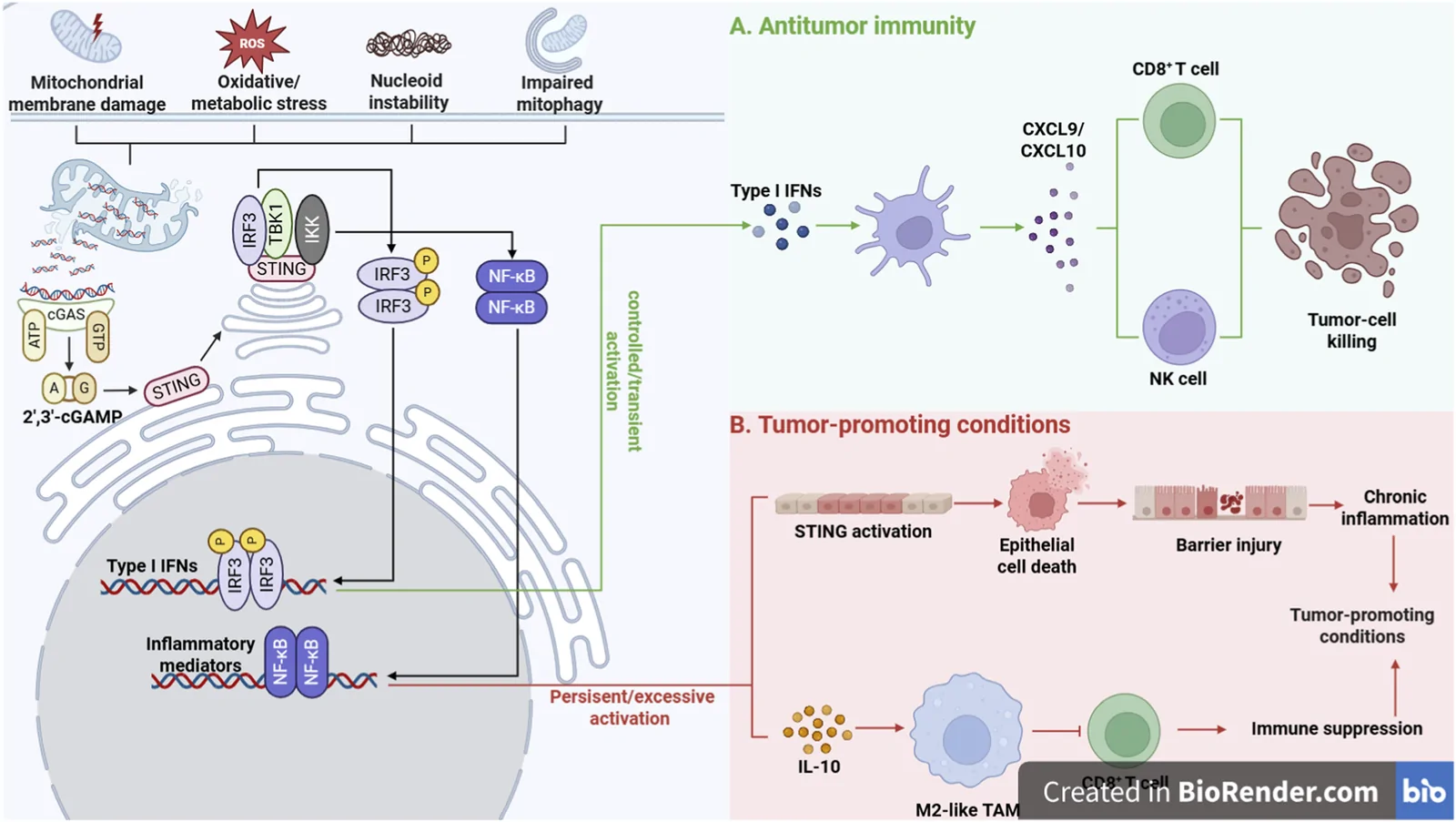
The mtDNA–cGAS–STING axis in colorectal cancer: transient versus persistent signaling and divergent biological outcomes. **(A)** Antitumor immunity. **(B)** Tumor-promoting conditions. Created in BioRender. G, H. (2026) https://BioRender.com/keyyuko.

These cell-specific effects indicate that pathway activity should be interpreted according to the cellular compartment in which signaling occurs. A modest signal concentrated in dendritic cells or other antigen-presenting cells may support lymphocyte recruitment, whereas comparable or stronger pathway activation in normal epithelium or suppressive myeloid populations may contribute to tissue injury or immunosuppression. Cell-resolved assessment of pathway activity is therefore more informative than bulk-tumor STING expression or IFN-I measurements alone (Figure 1).

## 3 Bidirectional roles of the mtDNA-cGAS-STING signaling axis in colorectal cancer

### 3.1 Initiation of antitumor immune responses

In CRC with insufficient immune-cell infiltration, activation of the cGAS-STING pathway may restore or enhance antigen presentation and effector lymphocyte recruitment. STING-induced IFN-I can increase the antigen-presenting capacity of dendritic cells and generate chemotactic signals that facilitate the entry of cytotoxic lymphocytes into tumor tissues (Kwon and Bakhoum, 2020). Therapeutic benefit therefore depends not only on pathway activation within tumor cells but also on the ability of antigen-presenting cells to receive and translate the resulting innate immune signal.

Cytosolic DNA in CRC can originate from sources other than mitochondria. DNA-repair defects can generate nuclear-derived cytosolic DNA and activate innate immune signaling. Inhibition of the endonuclease EEPD1 increases cytosolic DNA by impairing homologous recombination repair (Huo et al., 2026). Myeloid-cell state further influences how cytosolic DNA sensing is converted into adaptive immunity. Macrophage-specific loss of secreted phosphoprotein 1 (SPP1) activates the ROS-DNA fragment-cGAS-STING-STAT1-CXCL9/10 cascade and increases CD8^+^ T-cell infiltration (Wang et al., 2026b). These mechanisms illustrate that both DNA source and the responding immune compartment influence pathway output.

Mismatch repair deficiency provides another source of cytosolic DNA. dMMR-associated DNA fragments can activate antitumor innate immunity (Mosley et al., 2026), and dMMR/MSI-H CRC exhibits greater endogenous cGAS-STING activity together with features of an immune-inflamed tumor microenvironment (Kaneta et al., 2022). mtDNA-dependent mechanisms should therefore be distinguished from nuclear-DNA-driven pathway activation, particularly in tumors with substantial genomic instability.

This distinction defines an important evidentiary boundary for the present review. Increased cGAS-STING activity alone cannot by itself establish mtDNA-origin activation. An mtDNA-specific mechanistic interpretation requires direct evidence of mitochondrial DNA release or an experimental approach capable of distinguishing mtDNA from nuclear cytosolic DNA. This issue is especially relevant in dMMR/MSI-H tumors, chromosomal-instability settings, and after genotoxic therapy, where nuclear DNA may provide a substantial competing pathway input.

### 3.2 Chronic inflammation, immunosuppression, and treatment resistance

When STING signaling is sustained or occurs predominantly in normal intestinal epithelium, IFN-I- and NF-κB-associated responses may shift from transient immune activation toward chronic inflammation. In inflammation-associated CRC, STING can contribute to mucosal defense while also promoting tumor initiation or progression under conditions of persistent tissue injury (Qu J. et al., 2026). Evidence from inflammatory bowel disease-associated CRC further emphasizes that the inflammatory state of the intestinal microenvironment is an important determinant of therapeutic outcome (Chen et al., 2025).

STING activation can also induce adaptive immune resistance. Upregulation of PD-L1 in tumor cells can suppress activated T-cell responses through the PD-1/PD-L1 axis, thereby limiting the durability of STING-mediated immune activation (Lin R. et al., 2026). This provides a mechanistic rationale for combining pathway activation with immune checkpoint blockade.

Immunosuppressive effects may also arise from altered downstream signal routing. MIIP downregulation increases cytosolic double-stranded DNA and activates the STING-NF-κB2-IL-10 axis, promoting M2-like macrophage polarization and metastasis-associated phenotypes (Chen et al., 2026). This finding demonstrates that detectable STING activation does not necessarily indicate an IFN-I-dominant antitumor response.

The tumor stroma can further suppress pathway competence. Co-culture with primary cancer-associated fibroblasts (CAFs) reduces endogenous cGAS and STING protein expression in CRC cells (Kanoda et al., 2025). Mitochondria-dependent tumor-stromal reprogramming has also been associated with treatment resistance, although the mechanisms linking stromal mitochondrial metabolism directly to cGAS-STING regulation in CRC remain incompletely defined (Li Y. et al., 2026). Tumor-cell STING abundance alone is therefore unlikely to capture pathway activity across the entire tumor microenvironment.

Mitochondrial quality control provides another route to immune escape. OPA3 suppresses mtDNA stress-mediated cGAS-STING signaling and promotes CRC progression (Yin et al., 2025). LRRC75A-AS1-ORF3-mediated mitophagy removes damaged mitochondria and attenuates immunogenic STING responses (Wu Q. et al., 2025). These mechanisms reduce the availability of immunogenic mitochondrial signals rather than directly inhibiting downstream STING kinases.

Therapeutic pressure can produce additional negative feedback. Radiotherapy-induced cholesterol synthesis suppresses tumor-cell-intrinsic STING signaling and contributes to treatment resistance (Zhu L. et al., 2025). The immune consequences of radiotherapy therefore depend on the balance between DNA- or mitochondrial-damage-associated pathway activation and therapy-induced metabolic suppression.

The opposing roles of cGAS-STING signaling in CRC are therefore better understood as context-dependent signaling states rather than a simple antitumor-versus-tumor-promoting switch. Persistent or spatially inappropriate activation can favor inflammatory or immunosuppressive outputs, whereas effective antitumor signaling requires a compatible combination of DNA input, pathway competence, responding cell type, and signal duration (Table 1).

**TABLE 1:** Context-dependent regulators and biological outcomes of the mtDNA-cGAS-STING axis in colorectal cancer.

Regulatory level	Key factor/event	Mechanism	Main cellular context	Biological consequence	Ref.
Mitochondrial membrane integrity	PLSCR3 loss	Disrupts inner-mitochondrial-membrane organization and promotes cytosolic mtDNA release	CRC cells	Enhances cGAS-STING activation and antitumor immunity	Ling et al. (2026)
Programmed cell death	GSDME	Induces mitochondrial damage and mtDNA-dependent IFN-β signaling	CRC cells; CD8^+^ T cells	Strengthens antitumor immune responses	Luo et al. (2024)
Mitochondrial permeabilization	BCL-2 inhibition/VDAC1 oligomerization	Promotes mtDNA release and subsequent STING activation	CRC cells	Induces STING-dependent antitumor immunity	Zhang et al. (2025a)
mtDNA epigenetic state	ACADS/SCAD	Reduces mtDNA methylation and facilitates cytosolic release	CRC cells	Enhances cGAS-STING-dependent antitumor immunity	Yang et al. (2026a)
Nucleoid stability	lncRNA 606938-TFAM axis	Regulates TFAM stability, oxidative stress, and mtDNA leakage	CRC cells	Promotes immunogenic pathway activation	Du et al. (2026)
Mitochondrial dynamics	DRP1 targeting	Restores radiotherapy-induced mitochondrial stress and mtDNA release	KRAS-mutant CRC	Enhances radiosensitivity and antitumor immunity	Tsai et al. (2026)
STING proteostasis	VCP/p97 inhibition	Stabilizes ER-retained STING and increases signaling persistence	CRC cells	Enhances STING activity and therapeutic response	Zhu et al. (2025a)
STING-independent cGAS activity	Metabolic reprogramming	Alters tumor metabolism independently of STING	CRC cells	Separates cGAS biology from canonical STING signaling	Wang et al. (2024)
Immune-cell/tumor-cell crosstalk	Immune-cell-intrinsic STING	Promotes ferroptosis in CRC cells	Immune cells and CRC cells	Amplifies local antitumor activity	Ding et al. (2026)
Myeloid reprogramming	DAI-triggered ferroptosis	Reprograms tumor-associated macrophages	CRC cells; TAMs	Favors an antitumor macrophage phenotype	Cheng et al. (2025)
Epithelial inflammatory response	cGAS-STING-mediated pyroptosis	Amplifies epithelial injury and inflammatory signaling.	Colonic epithelial cells	Promotes inflammation-associated CRC	Wang et al. (2026a)
Nuclear-DNA comparator	EEPD1 inhibition	Increases cytosolic DNA after defective homologous recombination	CRC cells	Activates cGAS-STING independently of mtDNA origin	Huo et al. (2026)
Myeloid immune output	Macrophage SPP1 loss	Activates ROS-DNA fragment-cGAS-STING-STAT1-CXCL9/10	TAMs; CD8^+^ T-cells	Increases CD8^+^ T-cell infiltration	Wang et al. (2026b)
Disease subtype	dMMR/MSI-H	Associated with greater endogenous pathway activation	Tumor and immune microenvironment	Supports an immune-inflamed phenotype	Kaneta et al. (2022)
Immunosuppressive signal routing	MIIP loss	Promotes STING-NF-κB2-IL-10 signaling	CRC cells; macrophages	Promotes M2-like polarization and metastatic phenotypes	Chen et al. (2026)
Stromal suppression	CAF-tumor interaction	Reduces tumor-cell cGAS/STING expression	CAFs and CRC cells	Weakens tumor-intrinsic DNA sensing	Kanoda et al. (2025)
Mitochondrial quality control	OPA3	Suppresses mtDNA-stress-mediated cGAS-STING signaling	CRC cells	Promotes tumor progression	Yin et al. (2025)
Mitophagy-mediated immune escape	LRRC75A-AS1-ORF3	Removes damaged mitochondria and reduces immunogenic mtDNA	CRC cells	Attenuates STING-mediated antitumor immunity	Wu et al. (2025b)
Therapy-induced metabolic feedback	Radiotherapy-induced cholesterol synthesis	Suppresses endogenous cGAS-STING activation after irradiation.	CRC cells	Contributes to radioresistance	Zhu et al. (2025c)
Tumor-intrinsic pathway competence	cGAS/STING expression in pMMR/MSS CRC	Associates pathway abundance with CD8^+^ T-cell infiltration	Tumor cells; CD8^+^ T cells	Potential prognostic and stratification value	Nakajima et al. (2023)

## 4 Therapeutic targeting of the mtDNA-cGAS-STING signaling axis in colorectal cancer

### 4.1 Direct STING agonism and delivery optimization

Direct STING agonism can bypass insufficient mtDNA release or weak upstream cGAS activation. This feature is attractive for tumors in which endogenous DNA sensing is inadequate. However, free STING agonists are limited by poor tumor accumulation, systemic inflammation exposure, and limited control over the duration of pathway activation. Delivery strategies have therefore focused on increasing intratumoral drug concentrations while reducing exposure of normal tissues (Singhai et al., 2026). In CRC, these challenges are further influenced by the intestinal barrier, peritoneal dissemination, and the local microbial environment (Li S.-Q. et al., 2026).

Nanomaterial-based systems provide several approaches to spatially restrict pathway activation. Tumor-responsive zinc-based nanoagonists can enhance local STING signaling under external stimulation (Hu et al., 2026), whereas zinc-manganese metal-organic frameworks combine cGAS-STING activation with immunogenic cell death (Zhu et al., 2026). Manganese-containing platforms can further enhance innate immune stimulation through chemodynamic and adjuvant effects (Yang Q. et al., 2026). These platforms are designed to concentrate pathway activation within the tumor while coupling STING signaling to additional tumor-damaging mechanisms.

Other delivery systems combine STING activation with photothermal, metabolic, or biomimetic interventions. Gold nanorod conjugates integrate STING agonism with photothermal therapy (Gao et al., 2026), whereas copper nanoparticles enhance cGAS-STING signaling through metabolic reprogramming (Tu et al., 2026). Salmonella-mimetic nanorobots can inhibit tumor glycolysis while activating cGAS-STING signaling during radioimmunotherapy (Liu et al., 2026). Locally administered *Bacillus* Calmette-Guerin hydrogel provides sustained NOD2/STING-mediated immune stimulation (Xiao et al., 2026), and STING-preactivated oncolytic hydrogel vaccines combine local oncolysis with innate immune priming (Guo et al., 2026).

Carrier design can also be adapted to specific disease settings. In models of peritoneal metastasis, manganese-alendronate nanomedicines promote oxidative and endoplasmic reticulum stress, immunogenic cell death, dendritic-cell maturation, and CD8^+^ T-cell responses (Qiu et al., 2026). The LPDAM photothermal platform uses ROS-induced mitochondrial injury to promote mtDNA release and employs Mn^2+^ to enhance cGAS-STING signaling (Pan et al., 2025). Other systems focus on the therapeutic cargo rather than the delivery environment. MIL-100(Fe)-based co-delivery integrates chemotherapy with cGAS-STING-directed immunotherapy (Wang P. et al., 2025), whereas dumbbell-shaped DNA nanostructures directly stimulate cGAS-STING signaling and enhance immune checkpoint therapy (Zhang Y. et al., 2025).

These approaches improve spatial control but do not eliminate the biological limitations of direct STING agonism. Increasing platform complexity can introduce additional challenges in pharmacokinetics, material clearance, manufacturing reproducibility, and safety assessment. Moreover, direct STING activation bypasses the DNA-sensing step and therefore does not preserve information about the origin of the upstream danger signal. These considerations favor localized, stimuli-responsive, or cell-targeted delivery over indiscriminate pathway-maximal stimulation.

### 4.2 Upstream mitochondrial stress interventions

Unlike direct STING agonism, upstream interventions act before pathway activation by increasing the availability of endogenous immunogenic mtDNA. This strategy may preserve cGAS-dependent DNA sensing while exploiting mitochondrial vulnerabilities in CRC cells.

Several CRC-associated mechanisms regulate mtDNA release through mitochondrial genome organization. Restoration of ACADS/SCAD expression reduces mtDNA methylation and promotes its translocation into the cytosol (Yang F. et al., 2026). The lncRNA 606938-TFAM axis similarly regulates mtDNA leakage by altering TFAM stability (Du et al., 2026). These approaches restore pathway input upstream of cGAS, although their therapeutic feasibility depends on target druggability and tumor specificity.

Mitochondrial membrane permeability and organelle dynamics provide additional intervention points. BCL-2 inhibition promotes VDAC1 oligomerization, mtDNA release, and STING-dependent antitumor immunity (Zhang W. et al., 2025). Targeting DRP1 can restore radiotherapy-induced mitochondrial stress and enhance mtDNA release in KRAS-mutant CRC (Tsai et al., 2026). These findings indicate that mitochondrial damage generated by pharmacological treatment or radiotherapy can be converted into an innate immune stimulus.

Existing drugs and bioactive compounds may also induce immunogenic mitochondrial stress. Lovastatin promotes mitochondrial oxidative stress and mtDNA release, followed by cGAS-STING-dependent apoptosis in CRC models (Huang et al., 2024).

Andrographolide induces VDAC-associated mitochondrial dysfunction and activates cGAS-STING signaling, accompanied by increased effector immune-cell infiltration and reduced regulatory T-cell abundance (Wu J. et al., 2025). Such approaches may provide opportunities for drug repurposing, although their mitochondrial effects are unlikely to be restricted exclusively to tumor cells.

Nutrient availability also influences mtDNA-dependent pathway activation. Aspartate deprivation disrupts nucleotide metabolism, promotes mtDNA damage and release, and increases sensitivity to cGAS-STING-targeted therapy (Liao et al., 2026). Serine depletion similarly activates mtDNA-dependent cGAS-STING signaling in CRC models (Saha et al., 2024). However, systemic metabolic interventions may affect immune cells and normal intestinal tissues in addition to tumor cells.

A major limitation of upstream mitochondrial intervention is its dependence on pathway competence. Increased mtDNA release will not necessarily produce productive antitumor immunity when cGAS or STING is deficient, epigenetically suppressed, or functionally rewired. Excessive mitochondrial damage may also injure normal tissues or sustain inflammatory signaling. Thus, the value of upstream intervention depends on tumor-selective mitochondrial stress, preserved downstream signaling, and appropriate control of the magnitude and duration of injury.

### 4.3 Combination with immunotherapy, radiotherapy, and chemotherapy

For MSS/pMMR CRC, STING activation may be more useful as an immune-priming strategy than as a stand-alone therapeutic endpoint. Innate immune activation can enhance antigen presentation and lymphocyte recruitment, but it can also induce adaptive immune resistance. A bifunctional STING agonist/PD-L1 inhibitor addresses both processes by combining pathway activation with blockade of PD-L1-mediated feedback (Lin R. et al., 2026).

Radiotherapy provides another opportunity to integrate DNA damage with innate immune activation. Radiation can generate cytosolic DNA and mitochondrial stress, but the resulting immune response is strongly influenced by tumor-intrinsic and microenvironmental factors. Targeting DRP1 restores radiotherapy-induced mtDNA release in KRAS-mutant CRC and enhances STING activation (Tsai et al., 2026). PI3Kδ/γ inhibition further enhances macrophage-dependent cGAS-STING-type I interferon signaling after radiotherapy (Kim and Kim, 2026). Mitochondrial p32 has also been associated with TBK1 phosphorylation and radiosensitivity in CRC (Wu Y. et al., 2025).

Additional regulatory mechanisms may modify radioimmune responses. C5aR1 and cGAS/STING signaling have been proposed to interact in CRC radiosensitivity, although direct clinical validation remains limited (Pham et al., 2026). Hybrid nanoadjuvants can also enhance cGAS-STING-type I interferon signaling during radioimmunotherapy (Jiang et al., 2026). These findings highlight the importance of treatment sequence, because DNA damage, innate immune priming, and checkpoint blockade should ideally occur within a compatible therapeutic window.

Local delivery may further improve combination treatment. *Bacillus* Calmette-Guerin hydrogel can sustain innate immune stimulation within tumors and enhance the activity of immune checkpoint blockade (Xiao et al., 2026). Other strategies strengthen T-cell recruitment or create a dMMR-like DNA stress state. Bimetallic nanobombs combine DNA fragmentation with Mn^2+^/Co^2+^-mediated STING activation and enhance bispecific T-cell engager-mediated responses in preclinical models (Mu et al., 2026). PRMT5 inhibition combined with CPT-11 reduces PMS2, generates a dMMR-like cytosolic DNA state, and improves responses to anti-TIGIT therapy (Zhu J. et al., 2025).

Chemotherapy can also be incorporated into STING-directed treatment by exploiting DNA and mitochondrial injury. A curcumin/oxaliplatin co-loaded hydroxyapatite platform induces nuclear DNA damage, Ca^2+^ overload, mitochondrial injury, and mtDNA release, thereby enhancing cGAS-STING activation (Xiao et al., 2024). An oxaliplatin-artesunate conjugate similarly enhances antitumor immunity in CRC models (Tan et al., 2026).

The principal challenge of combination therapy is therefore not the number of therapeutic components, but their biological coordination. Genotoxic treatment should generate productive immune priming without producing persistent inflammatory signaling, while checkpoint inhibition should coincide with the period of effective antigen presentation and lymphocyte recruitment.

### 4.4 Metabolic and gut microbiota modulation

CRC develops within a tissue environment strongly influenced by nutrient availability, host metabolism, and the gut microbiota. These factors shape the basal metabolic and inflammatory state of tumor and immune cells and may therefore modify responsiveness to cGAS-STING-directed therapy.

Engineered probiotics that deplete methionine can activate STING signaling and enhance the efficacy of anti-PD-L1 therapy (Sun et al., 2026). This approach links nutrient restriction with innate immune activation, although its effect may vary according to microbiota composition and dietary status.

Microbiota-derived metabolites may also modify pathway activity. Specific bile acids can induce cGAS-STING-dependent type I interferon responses (He et al., 2026), suggesting that microbial metabolites can influence the response threshold of tumor and immune cells rather than serving only as background components of intestinal inflammation.

Microbiota-directed delivery systems provide another strategy. Porphyrin nanohybrids targeting *Fusobacterium* nucleatum can alter local microbiota composition while remodeling the tumor immune microenvironment (Liang et al., 2025). Microbiota-based biomarker studies further support the possibility of patient stratification according to dysbiosis patterns (Yang L. et al., 2026). Broader evidence links microbiota-associated inflammation, metabolism, biofilms, and cGAS-STING-related signaling to CRC development (Bachir et al., 2026).

These approaches face substantial translational variability. Microbiota composition is influenced by geography, diet, antibiotics, other medications, and sampling methods. Metabolic interventions likewise affect both tumor and host physiology. Consequently, metabolism- and microbiota-based strategies should be regarded as indirect modulators of the pathway rather than mtDNA-specific therapies.

### 4.5 Clinical rationale for mtDNA-origin upstream targeting versus direct STING agonism

A potential advantage of upstream mtDNA-origin targeting is not that cGAS can biochemically distinguish mitochondrial DNA from other double-stranded DNA. Rather, selectivity must arise at the intervention level. Therapeutic perturbation of mitochondrial membrane integrity, nucleoid stability, dynamics, or metabolism may preferentially increase mtDNA release within stressed tumor cells. Selective induction of mtDNA release while limiting genomic DNA damage has therefore been proposed as a potential strategy to optimize STING activation (Lu et al., 2026).

This approach differs fundamentally from direct STING agonism. Direct agonists bypass upstream DNA sensing and can activate STING even when endogenous mtDNA release or cGAS activity is insufficient. In contrast, mtDNA-origin interventions retain endogenous cGAS as a biological gate. If mitochondrial stress can be preferentially induced within the tumor, this architecture may generate more localized or transient cGAMP production and reduce direct exposure of normal immune and epithelial cells to exogenous STING agonists.

However, this proposed advantage remains hypothetical at the clinical level. No evidence currently demonstrates that mtDNA-origin intervention reduces systemic inflammation or off-target toxicity in patients with CRC. Normal intestinal cells may also release mtDNA under mitochondrial stress, and tumors with deficient cGAS-STING signaling may remain unresponsive despite increased mtDNA release.

mtDNA-origin signaling must also be distinguished from nuclear-DNA-driven and noncanonical STING activation. This distinction is particularly important during radiotherapy and chemotherapy, which can simultaneously generate mitochondrial injury and nuclear DNA damage. Pharmacodynamic studies should therefore establish DNA origin, cGAMP generation, STING activation, and downstream branch-specific responses rather than using increased bulk STING activity or IFN-I production alone as evidence of mtDNA-specific target engagement (Table 2).

**TABLE 2:** Representative therapeutic strategies targeting the mtDNA-cGAS-STING axis in colorectal cancer.

Strategy	Representative intervention	Main advantage	Main limitation	Ref.
Localized STING activation	TME/NIR-responsive zinc nanoagonist	Enhances local STING activation while limiting nonspecific exposure	Requires external irradiation and validation of long-term material safety	Hu et al. (2026)
Metal-enhanced STING activation	Zn-Mn metal-organic framework	Combines STING activation with immunogenic cell death	Metal clearance, pharmacokinetics, and manufacturing complexity	Zhu et al. (2026)
Photothermal-STING combination	STING agonist-gold nanorod conjugate	Synchronizes local tumor injury with innate immune activation	Limited light penetration and complex pharmacokinetics	Gao et al. (2026)
Biomimetic radioimmunotherapy	Salmonella-mimetic nanorobots	Couples glycolytic inhibition with cGAS-STING activation during radiotherapy	Biosafety, biodistribution, and manufacturing reproducibility	Liu et al. (2026)
Local immune priming	BCG hydrogel	Provides sustained local innate stimulation and improves checkpoint responsiveness	Requires control of local inflammation and release kinetics	Xiao et al. (2026)
mtDNA-inducing Photothermal therapy	LPDAM platform	Promotes mitochondrial damage while enhancing cGAS-STING signaling	Multicomponent formulation and light-dependent delivery	Pan et al. (2025)
Direct DNA-mediated activation	Dumbbell-shaped DNA nanostructure	Directly activates cGAS-STING and enhances checkpoint therapy	Nuclease stability and possible systemic DNA sensing	Zhang et al. (2025b)
Upstream mitochondrial stress	BCL-2 inhibition/VDAC1 oligomerization	Induces endogenous mtDNA release while retaining cGAS-dependent sensing	Mitochondrial toxicity may not be tumor selective	Zhang et al. (2025a)
Mitochondrial dynamics targeting	DRP1 targeting	Restores radiotherapy-induced mtDNA release in KRAS-mutant CRC	Requires genotype selection and optimized treatment timing	Tsai et al. (2026)
Drug repurposing	Lovastatin	Induces mitochondrial oxidative stress and endogenous mtDNA release	Effective dose and off-target metabolic effects require evaluation	Huang et al. (2024)
Metabolic sensitization	Aspartate deprivation	Increases mtDNA damage/release and sensitizes tumors to pathway activation	May affect normal and immune-cell metabolism	Liao et al. (2026)
Checkpoint-feedback blockade	Bifunctional STING agonist/PD-L1 inhibitor	Combines innate immune priming with simultaneous checkpoint inhibition	Agonist exposure and inflammatory toxicity require careful control	Lin et al. (2026a)
Myeloid radioimmune modulation	Dual PI3Kδ/γ inhibition	Enhances macrophage-dependent STING-IFN-I signaling after radiotherapy	Myeloid selectivity and broader immune effects remain uncertain	Kim and Kim (2026)
dMMR-like immune sensitization	PRMT5 inhibitor plus CPT-11	Generates cytosolic DNA stress and improves checkpoint sensitivity in MSS CRC	Genotoxicity and treatment sequence require optimization	Zhu et al. (2025b)
Chemo-mitochondrial activation	Curcumin/oxaliplatin hydroxyapatite platform	Combines nuclear DNA damage, mitochondrial injury, mtDNA release, and STING activation	Carrier complexity and mixed nuclear/mitochondrial DNA inputs	Xiao et al. (2024)
Metabolism-microbiota modulation	Methionine-depleting engineered probiotic	Couples nutrient restriction with STING activation and PD-L1 blockade	Strong dependence on microbiota and dietary context	Sun et al. (2026)
Microbiota-targeted nanotherapy	*Fusobacterium* nucleatum-targeted porphyrin nanohybrid	Combines microbiota remodeling with cGAS-STING-associated immune activation	Interpatient microbiome variability limits reproducibility	Liang et al. (2025)

## 5 Discussion and future perspectives

### 5.1 Defining the therapeutic window for precise pathway modulation

The major translational challenge is not simply to activate cGAS-STING signaling, but to determine where, when, and to what extent the pathway should be activated. Productive signaling requires a balance between sufficient innate immune priming and avoidance of persistent inflammatory or cytotoxic responses.

This problem is particularly relevant to CRC because tumor cells, macrophages, T cells, fibroblasts, and intestinal epithelial cells may respond differently to the same stimulus. Pathway evaluation should therefore move beyond whole-tumor STING expression or IFN-I measurements. Cell-specific localization, STING abundance and trafficking, TBK1/IRF3 activation, NF-κB-associated outputs, autophagy or mitophagy status, and metabolic status can provide more mechanistically informative readouts.

Spatial and temporal control should also become central to therapeutic design. Local administration, stimuli-responsive delivery, transient mitochondrial stress, and reversible pathway modulation may provide a wider therapeutic window than persistent systemic stimulation. Combination regimens additionally require treatment sequencing so that innate immune priming coincides with antigen presentation and checkpoint blockade.

For mtDNA-origin interventions, pharmacodynamic assessment should also verify the source of cytosolic DNA. Increased STING activity alone cannot establish mtDNA-specific target engagement. Source-resolved DNA measurements, together with cGAMP and cell-specific downstream signaling, will be necessary to distinguish productive mtDNA-driven activation from nuclear-DNA-related or noncanonical signaling.

### 5.2 Establishing patient stratification and biomarker systems

Molecular subtype provides an initial framework for patient selection, but mismatch repair status alone is unlikely to predict response to mtDNA-cGAS-STING-directed therapy. The clinical relevance of this distinction is illustrated by the durable benefit of immune checkpoint blockade in MSI-H/dMMR (André et al., 2020). MSI-H/dMMR and MSS/pMMR tumors also differ in endogenous cGAS-STING activity and immune infiltration (Kaneta et al., 2022). Within MSS/pMMR CRC, tumor-cell cGAS/STING expression has also been associated with CD8^+^ T-cell infiltration and clinical outcome (Nakajima et al., 2023). Patient stratification should therefore integrate molecular subtype with pathway competence, cellular localization, and microenvironmental composition.

Circulating mtDNA provides a potential minimally invasive biomarker. The broader biomarker landscape in CRC includes circulating cell-free mtDNA and other mitochondrial genome-related signals with potential relevance to liquid biopsy (Koo et al., 2025). Cell-free mtDNA fragmentation profiles have been investigated for early CRC detection (Wang S. et al., 2025). However, circulating mtDNA measurements are sensitive to sample collection, processing, analytical methods, and tumor stage (Linke et al., 2023). Standardized pre-analytical and analytical procedures will therefore be required before these measurements can be used reliably for longitudinal treatment monitoring.

Tissue-based mitochondrial markers may provide complementary information. mtDNA copy number shows subtype-dependent associations with immune infiltration and has been investigated as a potential guide for adjuvant chemotherapy in dMMR CRC (Chen et al., 2024b; 2024a). mtDNA methylation may also have diagnostic or prognostic potential (Wang Q. et al., 2025), whereas mtDNA mutational signatures currently remain more useful for mechanistic investigation than for routine therapeutic selection (Guo et al., 2023).

Mitochondrial and metal-homeostasis alterations in early lesions may provide additional information about pathway state. Reduced SLC30A10 expression and altered manganese homeostasis in colorectal adenomas have been associated with cGAS-STING activation (Qu T. et al., 2026). Changes in telomere length and mtDNA copy number across the adenoma-carcinoma sequence may also reflect early mitochondrial alterations during colorectal tumor development (Valickova et al., 2026). These findings are currently more relevant to biological and risk-stratification research than to direct therapeutic decision-making.

Single-cell approaches may further help resolve intratumoral mitochondrial heterogeneity. mtDNA heteroplasmy has been detected among individual CRC cells (Almeida et al., 2022), and dynamic mtDNA alterations in CRC stem-cell populations have been associated with stemness and treatment response (Shakhpazyan et al., 2024). These observations remain exploratory and require validation in large cohorts.

No biomarker panel has yet been prospectively validated specifically for mtDNA-cGAS-STING-directed therapy. Future biomarker development should therefore combine baseline molecular stratification with dynamic pharmacodynamic measurements capable of resolving DNA origin, pathway competence, and the cellular compartment in which signaling occurs.

### 5.3 Advancing preclinical evidence toward clinical translation

Most approaches targeting the mtDNA-cGAS-STING axis remain at the cellular or animal-model stage. This is particularly true for mitochondrial stress interventions, multifunctional nanoplatforms, and microbiota-based therapies.

Patient-derived organoids can reproduce important tumor-cell-intrinsic features but lack a complete immune and stromal microenvironment. Humanized mouse models provide additional information on immune responses but do not fully reproduce patient-specific microbiota, chronic intestinal inflammation, or metabolic conditions. No single model can therefore capture all components of the pathway.

Future studies should match experimental design to the therapeutic entry point. Upstream mitochondrial interventions require direct confirmation of mtDNA release and cGAS dependence. Direct STING agonists require cell-specific pharmacokinetic and downstream signaling measurements. Combination regimens require longitudinal sampling across the immune-priming and checkpoint-blockade windows.

Integration of patient-derived organoids with autologous immune and stromal cells, spatial and single-cell profiling, and longitudinal liquid biopsy may provide the necessary temporal and compartmental resolution. Early-phase clinical studies should also incorporate biomarker-based patient selection and pharmacodynamic measurements rather than relying only on maximum tolerated dose or bulk pathway expression.

For MSS/pMMR CRC in particular, a central clinical question is whether controlled innate immune priming can convert immune-excluded tumors into a state responsive to checkpoint blockade. Such trials should evaluate antitumor efficacy, inflammatory toxicity, and the cellular compartment in which STING activation occurs.

## 6 Limitations

Several limitations should be considered when interpreting the evidence summarized in this review. Most therapeutic studies involving STING agonism, mitochondrial stress, nanodelivery, or microbiota-directed interventions remain preclinical, and their efficacy and safety cannot yet be directly extrapolated to patients with CRC. This limitation is particularly relevant to complex nanomaterials and mitochondrial interventions, for which pharmacokinetics, tissue selectivity, and long-term safety remain incompletely defined.

Interpretation is further complicated by uncertainty regarding the origin of cytosolic DNA. Mitochondrial damage or increased total cytosolic DNA is sometimes taken as indirect evidence of mtDNA involvement without formally excluding nuclear DNA. This is especially problematic in dMMR/MSI-H tumors, chromosomal-instability settings, and after radiotherapy or chemotherapy, where mitochondrial and nuclear DNA stress may occur simultaneously. Increased cGAS-STING activity alone therefore cannot establish an mtDNA-specific mechanism.

Current experimental approaches also provide limited resolution of pathway heterogeneity. STING activity is frequently assessed in bulk tissue or at a single time point, which can obscure differences in STING abundance, downstream branch activation, autophagic flux, metabolic state, and treatment response among tumor cells, immune cells, and normal intestinal epithelial cells. Species-specific differences in STING pharmacology and immune composition further complicate the translation of findings from animal models to humans.

Comparison across therapeutic studies remains difficult because experimental models, formulations, doses, treatment sequences, and endpoints vary substantially. These differences prevent reliable quantitative comparison of efficacy among current strategies. In parallel, no prospectively validated companion biomarker has yet been established to identify patients most likely to benefit from mtDNA-cGAS-STING-directed intervention.

The present review also has methodological limitations. It is a focused narrative review rather than a systematic review or meta-analysis, and no formal risk-of-bias assessment, exhaustive systematic database search, or quantitative evidence pooling was performed. The framework proposed here, which distinguishes mtDNA-origin input from noncanonical STING signaling and STING-independent cGAS functions, should therefore be regarded as an interpretive model that requires further validation through source-resolved and cell-resolved experimental studies.

## 7 Conclusion

The mtDNA-cGAS-STING axis provides a mechanistic link between mitochondrial stress, innate immune sensing, and the immune microenvironment in CRC. A central conceptual contribution of this review is the distinction among canonical mtDNA-cGAS-cGAMP-STING signaling, noncanonical STING activation, and STING-independent cGAS functions. Within this framework, biological outcome is determined not simply by pathway activation, but by the origin of cytosolic DNA, the route of activation, downstream signal routing, the responding cell type, and the spatial and temporal pattern of signaling.

From a therapeutic perspective, direct STING agonism and mtDNA-origin upstream intervention offer distinct advantages and limitations. Direct agonists can bypass inadequate endogenous DNA sensing but require strict control of dose and tissue exposure. Upstream mitochondrial interventions retain endogenous cGAS-dependent sensing and may couple pathway activation to tumor mitochondrial stress. Their proposed spatial and safety advantages, however, remain unproven clinically and dependent on sufficient tumor selectivity and preserved pathway competence.

Current evidence remains limited by its predominantly preclinical nature, incomplete discrimination between mitochondrial and nuclear DNA, heterogeneous experimental models, insufficient cell-specific longitudinal measurements, and the absence of validated companion biomarkers. These limitations argue against indiscriminate escalation of STING activity and instead support mechanistically guided and context-dependent pathway modulation.

Future studies should integrate mtDNA-origin tracing with spatial and single-cell analyses to identify the cellular sources and recipients of productive signaling. Therapeutic development should place greater emphasis on tumor-localized and reversible pathway modulation, with treatment timing guided by pharmacodynamic changes in innate immune priming and checkpoint responsiveness. These mechanistic and therapeutic advances should ultimately be tested in biomarker-stratified early-phase trials, particularly in MSS/pMMR CRC, using both antitumor efficacy and inflammatory toxicity as clinically relevant endpoints. Such studies will determine whether precision modulation of the mtDNA-cGAS-STING axis can progress from a compelling biological concept to a reproducible therapeutic strategy in CRC.
